# A subregion-based RadioFusionOmics model discriminates between grade 4 astrocytoma and glioblastoma on multisequence MRI

**DOI:** 10.1007/s00432-023-05603-3

**Published:** 2024-02-02

**Authors:** Ruili Wei, Songlin Lu, Shengsheng Lai, Fangrong Liang, Wanli Zhang, Xinqing Jiang, Xin Zhen, Ruimeng Yang

**Affiliations:** 1 Department of Radiology, the Second Affiliated Hospital, School of Medicine, South China University of Technology, GuangZhou, China; 2 School of Biomedical Engineering, Southern Medical University, GuangZhou, China; 3 School of Medical Equipment, Guangdong Food and Drug Vocational College, Guangzhou, China

**Keywords:** Glioblastoma, Astrocytoma, Algorithm, Radiomics, Magnetic Resonance Imaging

## Abstract

**Purpose:**

To explore a subregion-based RadioFusionOmics (RFO) model for discrimination between adult-type grade 4 astrocytoma and glioblastoma according to the 2021 WHO CNS5 classification.

**Methods:**

329 patients (40 grade 4 astrocytomas and 289 glioblastomas) with histologic diagnosis was retrospectively collected from our local institution and *The Cancer Imaging Archive* (TCIA). The volumes of interests (VOIs) were obtained from four multiparametric MRI sequences (T_1_WI, T_1_WI + C, T_2_WI, T_2_-FLAIR) using (1) manual segmentation of the non-enhanced tumor (nET), enhanced tumor (ET), and peritumoral edema (pTE), and (2) *K*-means clustering of four habitats (H_1_: high T_1_WI + C, high T_2_-FLAIR; (2) H_2_: high T_1_WI + C, low T_2_-FLAIR; (3) H_3_: low T_1_WI + C, high T_2_-FLAIR; and (4) H_4_: low T_1_WI + C, low T_2_-FLAIR). The optimal VOI and best MRI sequence combination were determined. The performance of the RFO model was evaluated using the area under the precision-recall curve (AUPRC) and the best signatures were identified.

**Results:**

The two best VOIs were manual VOI_3_ (putative peritumoral edema) and clustering H_34_ (low T_1_WI + C, high T_2_-FLAIR (H_3_) combined with low T_1_WI + C and low T_2_-FLAIR (H_4_)). Features fused from four MRI sequences (${F}_{seq}^{\mathrm{1,2},\mathrm{3,4}}$) outperformed those from either a single sequence or other sequence combinations. The RFO model that was trained using fused features ${F}_{seq}^{\mathrm{1,2},\mathrm{3,4}}$ achieved the AUPRC of 0.972 (VOI_3_) and 0.976 (H_34_) in the primary cohort (*p* = 0.905), and 0.971 (VOI_3_) and 0.974 (H_34_) in the testing cohort (*p* = 0.402).

**Conclusion:**

The performance of subregions defined by clustering was comparable to that of subregions that were manually defined. Fusion of features from the edematous subregions of multiple MRI sequences by the RFO model resulted in differentiation between grade 4 astrocytoma and glioblastoma.

**Supplementary Information:**

The online version contains supplementary material available at 10.1007/s00432-023-05603-3.

## Introduction

Glioblastoma (GBM) is the most aggressive and malignant adult brain tumor that has a poor prognosis (Singh et al. 2021). According to the 2016 edition of the World Health Organization (WHO) classification criterion, gliomas can be grouped as per both histopathologic appearance and well-established molecular parameters. The malignant GBM is categorized as isocitrate dehydrogenase (IDH) mutant and IDH wildtype (Aldape et al. 2015; Chen et al. 2017). However, these two IDH variants exhibit distinct biological characteristics and clinical prognosis. To this end, the latest WHO CNS5 makes it clear that GBM particularly refer to IDH wildtype gliomas owing to their intrinsic invasive nature. In doing so, the previous “GBM” are now separated into GBM (IDH wildtype) and grade 4 astrocytoma (IDH mutant) given that they are quite distinct from biological characteristics and clinical behavior (Gritsch et al. 2022; Louis et al. 2021; Wen and Packer 2021). Thus, preoperative differentiation between these two entities paves the way for more effective patient stratification, targeted therapeutics, and prediction of patient outcomes. Radiomics is an emerging method that can automatically provide a large number of quantitative image features from medical images. Therefore, exploring a novel radiomics model for noninvasive discrimination between grade 4 astrocytoma and GBM provides an important reference for doctors to choose treatment options, which is of great significance to clinical practice.

Clinical decision-making on high-grade gliomas is determined by molecular genetic signatures, of which IDH status is the most important (Dang et al. 2016; Han et al. 2020). Due to the invasive procedures and limited samples for histology as well as the expensive cost of DNA sequencing for IDH testing, MRI-based radiographical examination is the most suitable option for non-invasive identification of IDH status, as it demonstrated excellent diagnostic capabilities for predicting IDH genotypes. Moreover, radiomics signatures from conventional, as opposed to advanced, MRI sequences were sufficient (Zhao et al. 2020). Nevertheless, most research has focused on lower-grade gliomas and their findings are thus possibly inapplicable to the 2021 WHO CNS 5 standard, albeit some studies showed promising performance (Chang et al. 2018; Suh et al. 2019; Yu et al. 2017). Constructing radiomics models from conventional MRI sequences and using them for routine clinical use is an attractive alternative as it requires no advanced sequencing techniques. However, its effectiveness, as per WHO CNS 5 standards, needs to be validated.

Subregional analysis has shown that radiomic metrics are capable of identifying distinct subpopulations that are more aggressive and treatment-resistant by exploring imaging features across the whole tumor, whose first step is segmentation of the tumor into several subregions, e.g., necrosis, enhancing core and peritumoral edema, by neuroradiologists or using deep learning segmentation methods (Chen et al. 2019; Li et al. 2018a, b; Rudie et al. 2019; Suhail et al. 2023). An alternative is use of *clustering* algorithms,—this method is also known as ‘*habitat imaging*’—which generates functionally coherent subregions of the tumor (Gatenby et al. 2013; Juan-Albarracin et al. 2018; Kim et al. 2021). These two tumor subregion definition methods are not only yet to be compared for glioma classification, but also have unknown impact on subsequent radiomics modeling.

To address the above issues, we herein proposed an MRI-based multisequence feature fusion model, namely *RadioFusionOmics* (RFO), to discriminate between grade 4 astrocytoma and GBM using subregional radiomics signatures from conventional MRI sequences. Thus, the two specific goals of this study were: (i) to develop a subregion-based RFO model —which is designed for grade 4 gliomas — for the prediction of IDH genotype, and (ii) to determine the impact of the two subregion definition strategies — manual and clustering — on model performance.

## Methods

### Patients

**Fig. 1:**
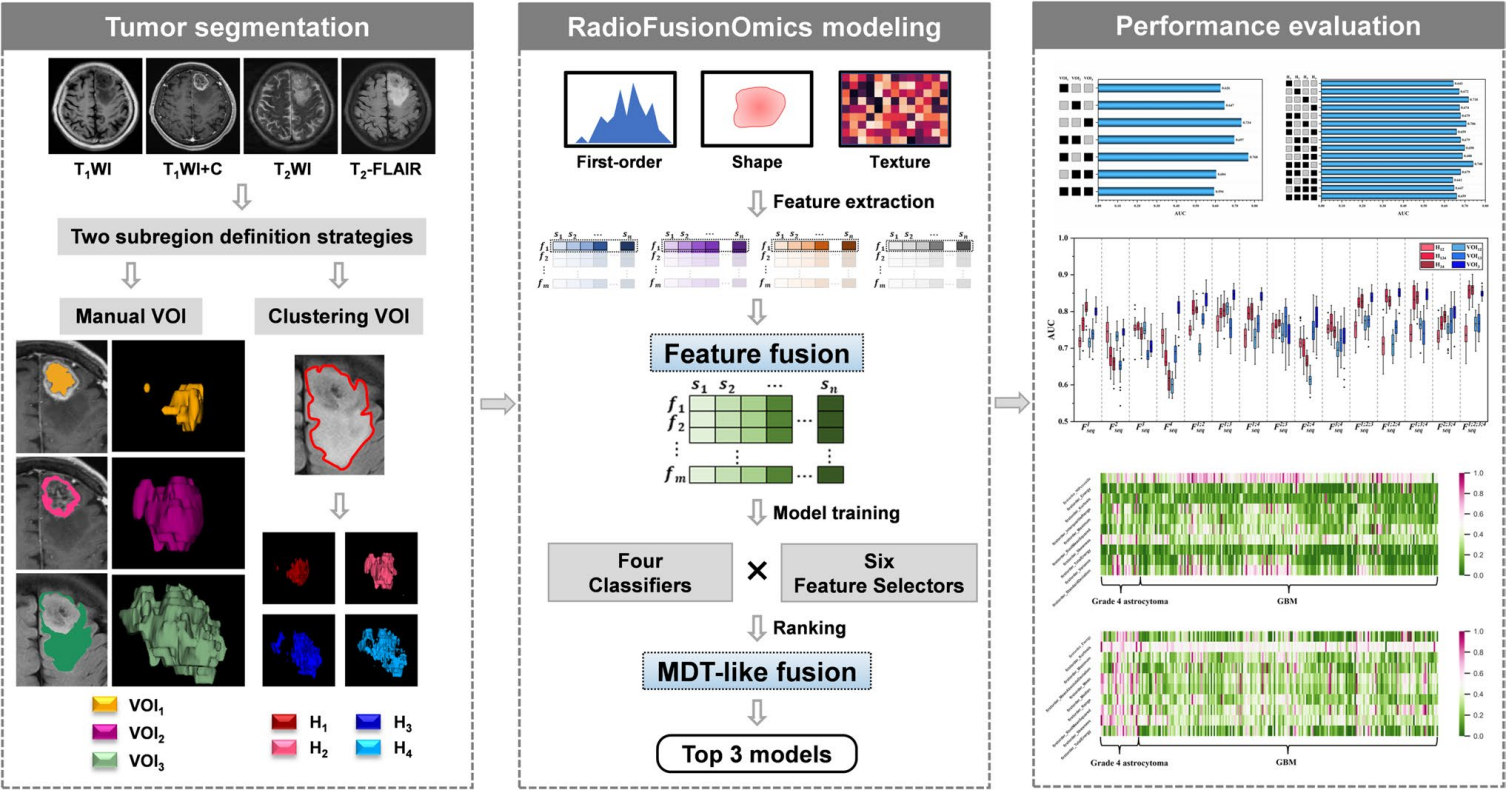
Flowchart of the subregion-based RadioFusionOmics pipeline used in this study, which includes segmentation, subregion definition, feature extraction and fusion, model development, and performance evaluation

Ethical approval for this retrospective study was granted by the local institutional review board, which waived the informed patient consent requirement. Patients who met the following criteria were enrolled: (1) age ≥ 18 years; (2) histologically reclassified as either grade 4 astrocytoma or GBM according to 2021 WHO CNS5; (3) had preoperative MRI examinations; (4) had no previous related treatments before MRI examination. Patients with a lesion with a pure solid or cystic component, or without enhancement, as well as poor MRI image quality were excluded. Thus, 259 patients (grade 4 astrocytoma, *n* = 36; GBM, *n* = 223) from our institution (Jan 2016 ~ Dec 2021), as well as 70 patients (grade 4 astrocytoma, *n* = 4; GBM, *n* = 66) from *The Cancer Imaging Archive* (TCIA) (https://www.cancerimagingarchive.net/; accessed on 25 November 2021) were enrolled. They were randomly divided into a primary (*n* = 230) and testing cohort (*n* = 99) (Fig. 1).

### Image acquisition

Preoperative MRI examinations were performed either on a 3.0 T (Verio, Siemens, Erlangen, Germany) or a 1.5 T MR scanner (Signa EXCITE HD, GE Healthcare, Milwaukee, WI, USA), both of which were equipped with 12‐channel head coils. Details of the MRI protocol were described in the Supplementary Materials. Four MRI sequences (T_1_WI, T_1_WI + C, T_2_WI, and T_2_-FLAIR) from each patient were aligned and resampled to the same geometry to T_1_WI using 3D slicer (https://www.slicer.org/).

### Subregion definitions

#### Strategy 1: Manual delineation

**Fig. 2:**
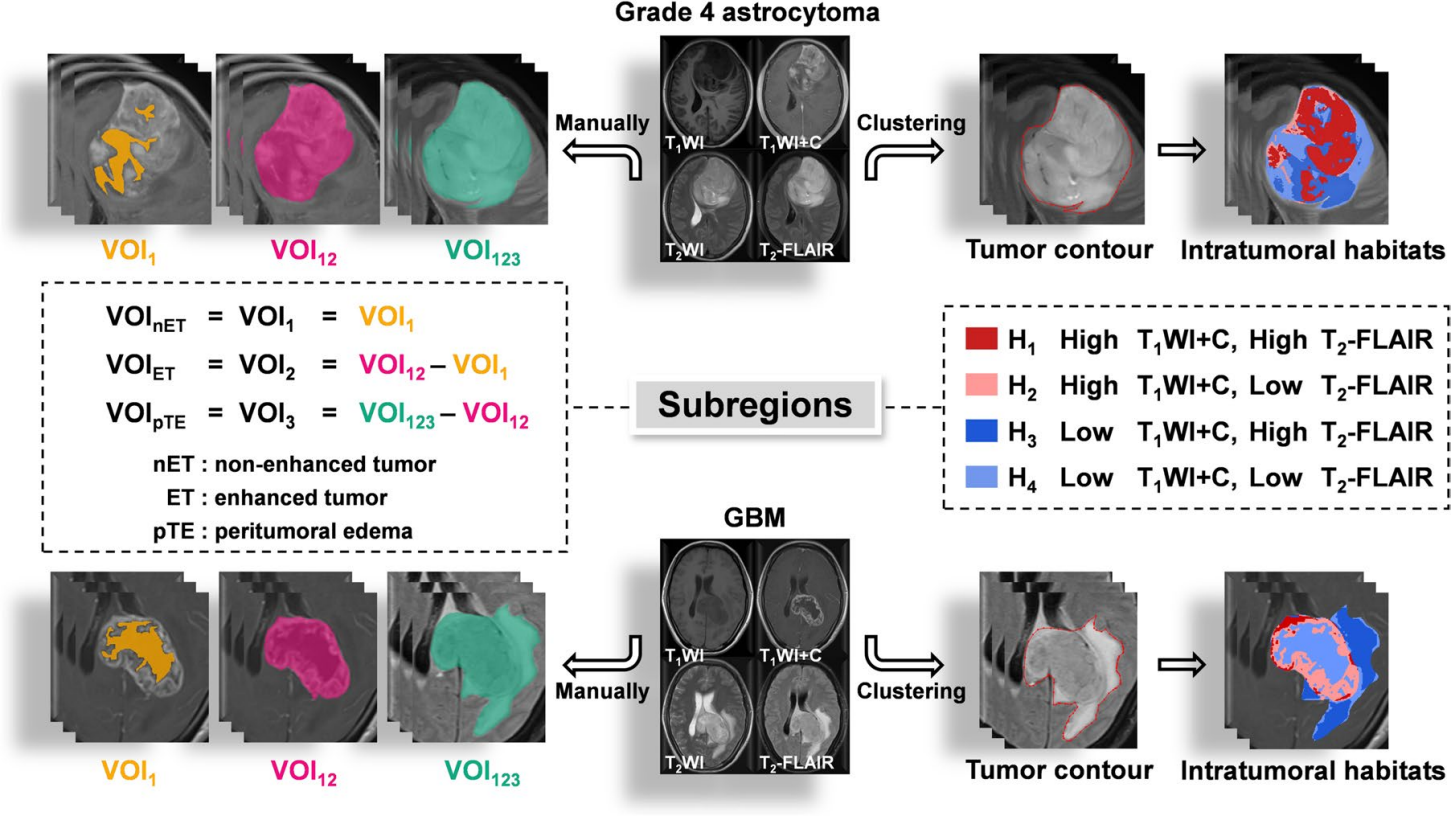
Representatives of two subregion definition strategies (manual (left) vs. K-means clustering (right)). Upper row: a 43‐year‐old female with pathologically confirmed grade 4 astrocytoma. Lower row: a 45‐year‐old female of GBM. For manual segmentation, the VOI_1_ (orange) and VOI_12_ (magenta) are delineated by T_1_WI + C images, while the VOI_123_ (green) is delineated by the T_2_-FLAIR image. For clustering, four clusters corresponded to H_1_: high T_1_WI + C, high T_2_-FLAIR (dark red); H_2_: high T_1_WI + C, low T_2_-FLAIR (light red); H_3_: low T_1_WI + C, high T_2_-FLAIR (dark blue); H_4_: low T_1_WI + C, low T_2_-FLAIR (light blue)

Three volumes of interests (VOIs) of the lesion — the non-enhanced tumor (nET, VOI_1_), the enhanced tumor (ET, VOI_2_), and the peritumoral edema (pTE VOI_3_) — were delineated by two board-certified investigators (R.L Wei and R.M Yang, with 6 and 15 years of expertise in radiological diagnosis, respectively) using the ITK‐SNAP software (http://www.itksnap.org). Inter-expert conformity was validated using Dice similarity coefficient. For those with Dice indexes > 0.9, the unanimous segmentation was the intersection of the two individual segmentations, while Dice indexes < 0.9, discrepancies on lesion boundary were resolved by further discussions to reach consensus. Different Boolean operations (e.g., “OR” or “NOT”) on the three VOIs defined seven regions of the tumor which were: VOI_1_ (putative non-enhanced tumor (nET)), VOI_2_ (putative enhanced tumor (ET)), VOI_3_ (putative peritumoral edema (pTE)), VOI_12_, VOI_13_, VOI_23_, and VOI_123_ (Fig. 2).

#### Strategy 2: Clustering algorithm

The largest VOI (i.e., VOI_123_), which encompassed the entire lesion, was used for subregion auto-clustering. Two regions of equal size in T_1_WI + C and T_2_-FLAIR (termed as ${{\text{VOI}}}_{123}^{{\text{T}}1{\text{WI}}+{\text{C}}}$ and ${{\text{VOI}}}_{123}^{{\text{T}}2-{\text{Flair}}}$) were extracted using the same VOI_123_ defined in T_2_-FLAIR (the four MRI sequences had been geometrically aligned). With intensities normalized between intra subject in ${{\text{VOI}}}_{123}^{{\text{T}}1{\text{WI}}+{\text{C}}}$ and ${{\text{VOI}}}_{123}^{{\text{T}}2-{\text{Flair}}}$, the intensity of each voxel $(x,y,z)$ in VOI_123_ was separated into high intensities and low intensities by K-means clustering algorithm (see Supplementary Materials) with a preset number of classes(n = 2), then the divided two regions of ${{\text{VOI}}}_{123}^{{{\text{T}}}_{1}{\text{WI}}+{\text{C}}}$ and ${{\text{VOI}}}_{123}^{{\text{Flair}}}$ were combined with Boolean operation (“AND”) into four clustering subregions H_1_, H_2_, H_3,_ and H_4_ such that ${{\text{VOI}}}_{123}={{\text{H}}}_{1}\cup {{\text{H}}}_{2}\cup {{\text{H}}}_{3}\cup {{\text{H}}}_{4}$. These subregions coincided with different radiographic metaphors for MRI sequences, i.e., (1) H_1_: high T_1_WI + C, high FLAIR (2) H_2_: high T_1_WI + C, low FLAIR (3) H_3_: low T_1_WI + C, high FLAIR and (4) H_4_: low T_1_WI + C, low FLAIR (Fig. 2).

### Feature extraction

Features (*n* = 109 features) were extracted from the seven manual and fifteen clustering VOIs of all four MRI sequences, using an open-source python package Pyradiomics (https://pyradiomics.readthedocs.io/en/stable/), the different types were first-order features (*n* = 19), shape features (*n* = 15) and texture features (*n* = 75).

### RFO modeling

We developed a *RadioFusionOmics* (RFO) model to discriminate between GBM and astrocytoma. The RFO model not only integrates radiomics information from different MRI sequences but also combines strengths of various classifiers via ensemble learning.

Briefly, using the feature level fusion of RFO, a feature-wise fusion scheme was performed by finding a transformation matrix $W$ to map the feature matrix $X$ with a given number of MRI sequences (e.g., *dimension* = 4) to a lower dimensional space (e.g., *dimension* = 1). By incorporating the class structure information (i.e., memberships of the training samples in class) in the calculation of the transformation matrix, the RFO reduced between-class correlations within the fused feature domain.

Using the model (or classifier) level fusion of RFO, different models were trained and ranked based on the fused features. The three best-performing models were identified and their predictions were unified via a multi-disciplinary team (MDT)-like fusion method, to create a consensus classification. Twenty-four models (6 feature selection methods: CMIM, DISR, ICAP, JMI, MIM, SPEC × 4 base classifiers: Extratrees Classifier, Gradient Boosting Classifier, eXtreme Gradient Boosting, Light Gradient Boosting Machine) were trained, and their discriminative performances were assessed and ranked using stratified fivefold cross-validations. The MDT-like model fusion was done by the weighted fusion method. Technical details regarding the RFO are in the Supplementary Materials. Data imbalance was addressed using the SMOTE (Synthetic Minority Over-Sampling Technique) method(García et al. 2012).

### Model evaluations

*Study 1*: Comparison of lesion VOIs

To identify the best lesion VOIs for discriminating between GBM and astrocytoma, the discriminative performances of features extracted from all MRI sequences using the seven manual (VOI_1_, VOI_2_, VOI_3_, VOI_12_, VOI_13_, VOI_23_, and VOI_123_) and fifteen clustering VOIs (H_1_, H_2_, H_3_, H_4_, H_12_, H_13_, H_14_, H_23_, H_24_, H_34_, H_123_, H_124_, H_134_, H_234_ and H_1234_) were compared.

*Study 2*: Comparisons of MRI sequences combinations

Different numbers and combinations of the four MRI sequences (${F}_{seq}^{\mathrm{1,2}}, {F}_{seq}^{\mathrm{1,3}},\dots , {F}_{seq}^{\mathrm{1,2},\mathrm{3,4}}$) were used as inputs for the RFO model that used the best lesion VOIs from Study 1. Then, their discriminative powers were compared and the best combinations of MRI sequences (for manual and clustering, respectively) for fusion were determined.

*Study 3*: Comparisons of manual vs. clustering VOIs

Comparisons were made between manual and clustering classification performances using either the best lesion VOIs and best MRI sequences or their combinations.

*Study 4*: Top features

The top-ranked features associated with grade 4 astrocytoma and their respective GBM classifications were identified by the RFO model and their discriminative capabilities were analyzed.

### Statistical analysis

Continuous variables were reported as mean ± SD, and categorical variables were reported as numbers and proportions. Normality of the data distribution was assessed by the Shapiro–Wilk test. The chi-square and Fisher’s exact tests were used to assess statistical significance of respective categorical variables in two and multiple groups. The Mann–Whitney U test was used to assess the statistical significance of non-normally distributed continuous variables. Statistical assessments of comparisons among the 15 feature types were performed using the Independent t-test to adjust the significance level in pairwise comparisons. For comprehensive evaluations of the classification performance on the imbalanced dataset, the area under the precision‐recall curves (AUPRC) were calculated for both primary and testing cohorts. All statistical analyses were conducted on SPSS version 20 (IBM). Two-tailed *p* < 0.05 was considered statistically significant.

## Results

### Patient characteristics

The patients’ demographic and radiological characteristics are summarized in Table 1. A total of 329 patients (51.70 ± 15.31 years) were enrolled —40 had grade 4 astrocytomas and 289 had GBM corroborating the low prevalence of mutations in the *IDH* gene of grade 4 astrocytomas (Cohen et al. 2013; Parsons et al. 2008). There were no significant differences in age, gender, tumor location, and tumor cross midline patterns between the primary and testing cohorts (*p* > 0.05), except for age and tumor dominant location within the primary cohort.

**Table 1:** Demographics and characteristics of the study cohort

Variables	Primary cohort (n = 230)		*p* (Intra)	Testing cohort (n = 99)		*P* (Intra)	*P* (Inter)
	Grade 4 astrocytoma (n = 28)	GBM (n = 202)		Grade 4 astrocytoma (n = 12)	GBM (n = 87)		
Gender			0.699			1.000	0.538
Male	15 (53.6)	116 (57.4)		7 (58.3)	53 (61.0)		
Female	13 (46.4)	86 (42.6)		5 (41.7)	34 (39.0)		
Age			0.001*			0.263	0.801
≤ 53	22 (78.6)	93 (46.0)		8 (66.7)	43 (49.4)		
> 53	6 (21.4)	109 (54.0)		4 (33.3)	44 (50.6)		
Dominant location			0.001*			0.058	0.696
Frontal lobe	22 (78.6)	67 (33.2)		8 (66.7)	25 (28.7)		
Parietal lobe	3 (10.7)	40 (19.8)		3 (25.0)	12 (13.8)		
Temporal lobe	0	41 (20.3)		1 (8.3)	22 (25.3)		
Occipital lobe	3 (10.7)	20 (9.9)		0	12 (13.8)		
Insular lobe	0	2 (1.0)		0	0		
Midline region	0	27 (13.4)		0	15 (17.2)		
Cerebellum	0	5 (2.5)		0	1 (1.1)		
Tumor cross midline			0.083			1.000	0.813
Yes	5 (17.9)	13 (6.4)		1 (8.3)	6 (6.9)		
No	23 (82.1)	189 (93.6)		11 (91.7)	81 (93.1)		

The median age was 53 years, and the patients were categorized as either above or below 53 years of age

Unless otherwise specified, the data is presented as frequencies, with percentages in parentheses. *p < 0.05

### Optimal VOI and best sequence combination

With strategy 1, seven manual VOIs (VOI_1_, VOI_2_, VOI_3_, VOI_12_, VOI_13_, VOI_23_, and VOI_123_) were individually generated for each MRI sequence and used in subsequent feature extraction. With strategy 2, subregions were coincided with different radiographic metaphors for MRI sequences, i.e., (1) H_1_: high T_1_WI + C, high T_2_-FLAIR; (2) H_2_: high T_1_WI + C, low T_2_-FLAIR; (3) H_3_: low T_1_WI + C, high T_2_-FLAIR; and (4) H_4_: low T_1_WI + C, low T_2_-FLAIR, resulting in fifteen clustering VOIs (H_1_, H_2_, H_3_, H_4_, H_12_, H_13_, H_14_, H_23_, H_24_, H_34_, H_123_, H_124_, H_134_, H_234_ and H_1234_).

**Fig. 3:**
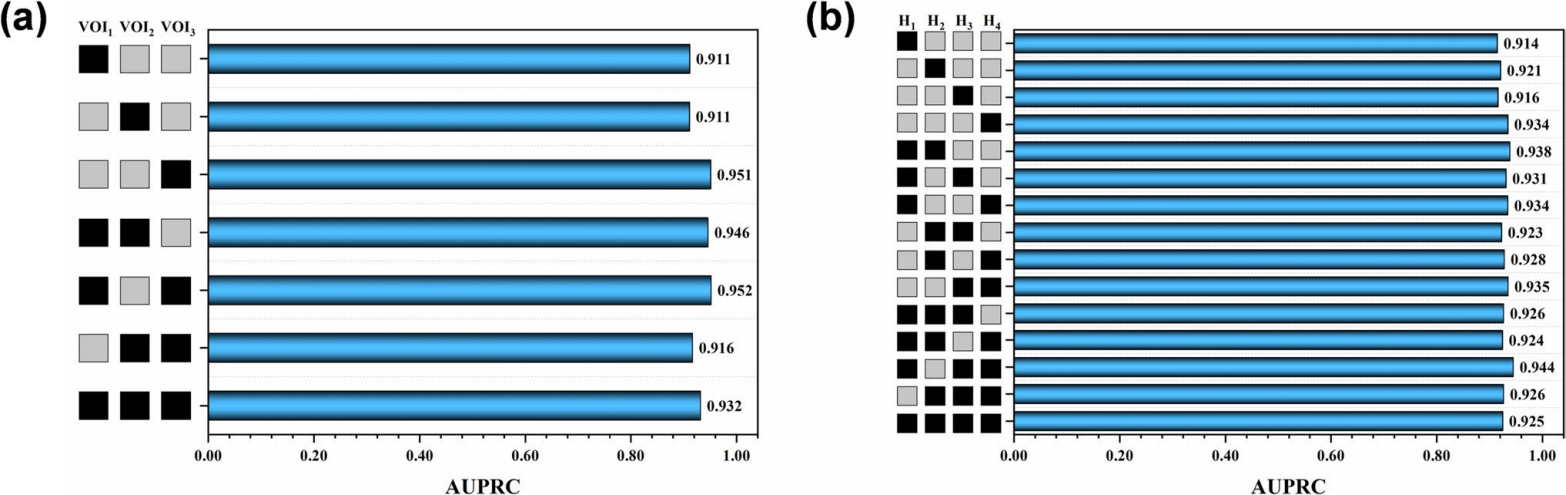
Prediction performances of **a** seven manual VOIs and **b** fifteen clustering VOIs

For each MRI sequence, we extracted radiomics features from 22 VOIs (7 manual + 15 clustering). Each type of feature (*n*_(manual)_ = 4 × 7 types; *n*_(clustering)_ = 4 × 15 types) was independently assessed in twenty-four models and evaluated by the five-fold cross-validation. As shown in Fig. 3A and B, image features from manual VOI_12_, VOI_13_ and VOI_3_, and clustering H_12_, H_134_ and H_34_ achieved the high AUPRC ( VOI_12_: maximum AUPRC = 0.969 on T_2_WI, mean AUPRC = 0.946 over all sequences; VOI_13_: maximum AUPRC = 0.972 on T_1_WI + C, mean AUPRC = 0.952 over all sequences; VOI_3_: maximum AUPRC = 0.975 on T_1_WI, mean AUPRC = 0.951 over all sequences; H_12_: maximum AUPRC = 0.966 on T_1_WI + C, mean AUPRC = 0.938 over all sequences; H_134_: maximum AUPRC = 0.962on T_2_WI, mean AUPRC = 0.944 over all sequences; H_34_: maximum AUPRC = 0.969 on T_1_WI, mean AUPRC = 0.935 over all sequences).

**Fig. 4:**
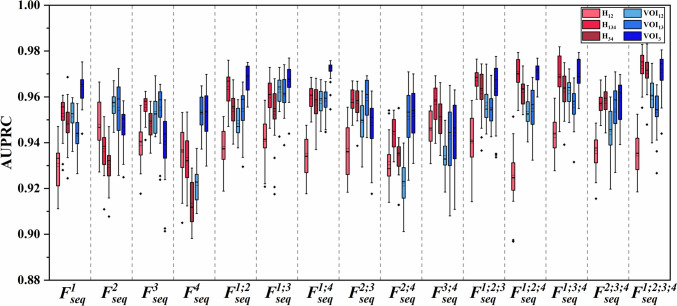
Performance comparisons of different combinations of the MRI sequences based on manual VOI_12_, VOI_13_, VOI_3,_ and clustering H_12_, H_134_, H_34_

Using the optimal subregions VOI_12_, VOI_13_, VOI_3_, and clustering H_12_, H_134_, H_34_, the features of either four MRI sequences (${F}_{seq}^{1}$, ${F}_{seq}^{2}$, ${F}_{seq}^{3}$, ${F}_{seq}^{4}$) or their fusions (${F}_{seq}^{1;2}, {F}_{seq}^{1;3},{F}_{seq}^{1;4}$, ${F}_{seq}^{2;3}$, ${F}_{seq}^{2;4}$, ${F}_{seq}^{3;4}$, ${F}_{seq}^{1;2;3}$, ${F}_{seq}^{1;2;4}$, ${F}_{seq}^{1;3;4}$, ${F}_{seq}^{2;3;4}$, ${F}_{seq}^{1;2;3;4}$) were compared using the RFO model in Fig. 4 and Table 2. From these comparisons, we observed that fusion of two/three/four MRI sequences generally outperformed that using a single sequence. This was consistent for both VOI_3_ and H_34_. Second, when fusing two/three MRI sequences, VOI_3_ generally achieved better performance than H_34_. Third, the highest mean AUPRC = 0.976 was obtained by H_34_ with fusion of all four MRI sequences (${F}_{seq}^{\mathrm{1,2},\mathrm{3,4}}$), and this was slightly better than its VOI_3_ counterpart that had a mean AUPRC = 0.972 (${F}_{seq}^{\mathrm{1,2},\mathrm{3,4}}$).

**Table 2:** Performances of the top manual and clustering VOIs (feature fusion of T_1_WI + T_1_WI + C + T_2_WI + T_2_-FLAIR in the RFO model) in the primary cohort

Primary cohort *N* = 230												
Top 3 models	Model 1	Model 2	Model 3	Mean	Model 1	Model 2	Model 3	Mean	Model 1	Model 2	Model 3	Mean
	Manual VOI_12_				Manual VOI_13_				Manual VOI_3_			
AUPRC	0.961	0.971	0.964	0.965	0.964	0.967	0.971	0.967	0.971	0.973	0.974	0.972
AUC	0.800	0.800	0.817	0.806	0.824	0.833	0.835	0.831	0.857	0.872	0.874	0.868
ACC	0.817	0.781	0.781	0.793	0.732	0.823	0.798	0.784	0.817	0.835	0.826	0.826
SPE	0.650	0.550	0.600	0.600	0.700	0.650	0.700	0.683	0.793	0.793	0.793	0.793
SEN	0.836	0.807	0.802	0.815	0.736	0.843	0.810	0.796	0.822	0.842	0.832	0.832
F1-SCORE	0.871	0.862	0.869	0.867	0.836	0.886	0.871	0.864	0.877	0.897	0.891	0.888
PRECISION	0.947	0.940	0.940	0.942	0.950	0.948	0.959	0.952	0.953	0.960	0.965	0.959
RECALL	0.836	0.807	0.802	0.815	0.736	0.843	0.810	0.796	0.822	0.842	0.832	0.832
	Clustering H_12_				Clustering H_134_				Clustering H_34_			
AUPRC	0.949	0.955	0.951	0.952	0.966	0.969	0.971	0.969	0.979	0.974	0.976	0.976
AUC	0.787	0.805	0.809	0.800	0.843	0.845	0.851	0.846	0.890	0.883	0.879	0.884
ACC	0.800	0.839	0.826	0.822	0.874	0.887	0.848	0.870	0.878	0.857	0.870	0.868
SPE	0.607	0.673	0.647	0.642	0.680	0.720	0.647	0.682	0.647	0.647	0.753	0.682
SEN	0.827	0.862	0.852	0.847	0.901	0.911	0.876	0.896	0.911	0.886	0.886	0.894
F1-SCORE	0.879	0.904	0.896	0.893	0.926	0.934	0.910	0.923	0.929	0.916	0.923	0.923
PRECISION	0.938	0.951	0.945	0.945	0.953	0.958	0.947	0.953	0.948	0.947	0.962	0.952
RECALL	0.827	0.862	0.852	0.847	0.901	0.911	0.876	0.896	0.911	0.886	0.886	0.894

**Fig. 5:**
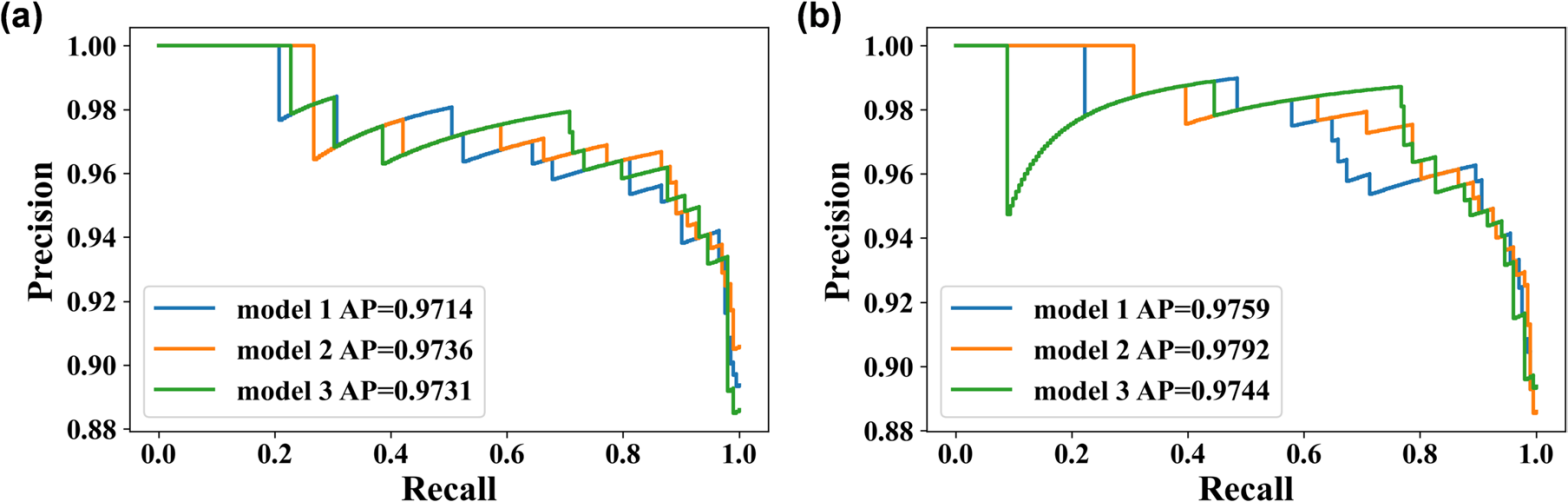
The precision-recall curve of the top-ranked models of **a** manual VOI_3_ and **b** the clustering H_34_

We used the best sequence fusion ${F}_{seq}^{\mathrm{1,2},\mathrm{3,4}}$ as well as optimal subregions (VOI_3_ and H_34_) for further independent evaluations of the testing cohort (Table 3, Fig. 5). In RFO, models were ranked during the training stage, and the three best-performing models were identified and fused to yield a final classification system for use in the testing stage. For ${F}_{seq}^{\mathrm{1,2},\mathrm{3,4}}$+ H_34_, the RFO model achieved mean AUPRC = 0.974, ACC = 0.758, SEN = 0.759 and SPE = 0.750, whereas values for ${F}_{seq}^{\mathrm{1,2},\mathrm{3,4}}$+VOI_3_, were mean AUPRC = 0.971, ACC = 0.666, SEN = 0.644, SPE = 0.833 (*p* = 0.402).

**Table 3:** Performances of the manual VOI_3_ and the clustering H_34_ (feature fusion of T1WI + T1WI + C + T2WI + T2-FLAIR in the RFO model) in the testing cohort

Top 3 models	Model 1	Model 2	Model 3	Mean	Model 1	Model 2	Model 3	Mean	p(AUPRC)	P(AUC)
	Manual VOI_3_				Clustering H_34_					
AUPRC	0.974	0.966	0.973	0.971	0.975	0.975	0.971	0.974	0.402	0.023*
AUC	0.824	0.824	0.823	0.824	0.842	0.842	0.830	0.838		
ACC	0.737	0.545	0.717	0.666	0.778	0.778	0.717	0.758		
SPE	0.833	0.833	0.833	0.833	0.750	0.750	0.750	0.750		
SEN	0.724	0.506	0.701	0.644	0.782	0.782	0.713	0.759		
F1-SCORE	0.833	0.652	0.819	0.768	0.861	0.861	0.816	0.846		
PRECISION	0.982	0.956	0.984	0.974	0.958	0.958	0.954	0.957		
RECALL	0.724	0.506	0.701	0.644	0.782	0.782	0.713	0.759		

*p < 0.05

### Radiomics signatures

**Fig. 6:**
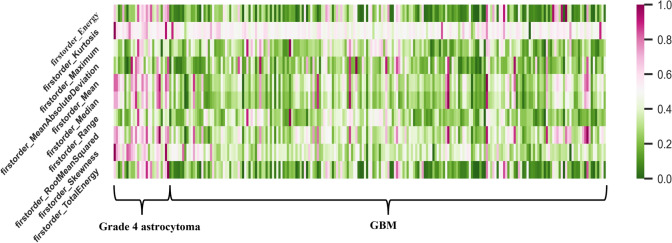
Heat maps of the ten best features

High-ranking features associated with discriminating between grade 4 astrocytoma and GBM were identified by the RFO models (using features of ${F}_{seq}^{\mathrm{1,2},\mathrm{3,4}}$+ H_34_). The heatmap of the ten highest-ranked features is shown in Fig. 6 and summarized in Table 4. The ten most frequently selected features included seven first-order statistics features (all with *p* < 10^–3^, except for the “MeanAbsoluteDeviation”) and three shape-based features (“Maximum2DDiameterRow” with *p* < 0.05). Using the average of the mean feature values of the two groups (i.e., “M” in Table 3) as the threshold for discrimination between grade 4 astrocytoma and GBM, all the first-order features demonstrated satisfactory discriminative capabilities.

**Table 4:** The ten most frequently selected features (using F_seq^1,2,3,4 + H34 in the RFO)

Category	Top10 Features	*p*-Value	M	(< M|> M)
First-order (n = 7)	Energy	< 10^–3^	0.14	Grade 4 astrocytoma (42.86% | 57.14%) GBM (70.30% | 29.70%)
	Kurtosis	< 10^–3^	0.13	Grade 4 astrocytoma (57.14% | 42.86%) GBM (78.22% | 21.78%)
	MeanAbsoluteDeviation	0.0801	0.47	Grade 4 astrocytoma (50.00% | 50.00%) GBM (70.79% | 29.21%)
	Mean	< 10^–5^	0.18	Grade 4 astrocytoma (35.71% | 64.29%) GBM (76.73% | 23.27%)
	Median	< 10^–6^	0.16	Grade 4 astrocytoma (35.71% | 64.29%) GBM (79.21% | 20.79%)
	RootMeanSquared	< 10^–4^	0.28	Grade 4 astrocytoma (28.57% | 71.43%) GBM (74.26% | 25.74%)
	Skewness	< 10^–6^	0.15	Grade 4 astrocytoma (42.86% | 57.14%) GBM (68.81% | 31.19%)
Shape (n = 3)	MajorAxisLength	0.0827	0.51	Grade 4 astrocytoma (53.57% | 43.43%) GBM (63.37% | 36.63%)
	Maximum2DDiameterRow	0.0180	0.37	Grade 4 astrocytoma (57.14% | 42.86%) GBM (65.84% | 34.16%)
	SphericalDisproportion	0.5515	2.07	Grade 4 astrocytoma (60.71% | 39.29%) GBM (60.40% | 39.60%)

‘M’ is the average of the mean feature value of the Grade 4 astrocytoma group and the mean feature value of the GBM group. ‘(< M |> M)’ represents the percentage of patients with a feature value less than or larger than the ‘M’ value. Values in bold indicate satisfactory discriminative capacities — ~ 70% of one group had larger or smaller feature values than the other group

## Discussion

The accurate prediction of IDH status in gliomas is crucial for guiding therapeutic decisions and management strategies. In this study, the proposed RFO model showed potential feasibility of IDH prediction for grade 4 gliomas which is not adequately predicted on the basis of histologic diagnosis. The fusion models from multiparametric MR images outperformed that from a single sequence. The comparison between two different subregion strategies revealed that voxel-wise habitats defined by the clustering procedure yielded a higher discriminative capability. Our results also implied that tumor edema may contain underlying heterogeneous metrics between grade 4 astrocytoma and GBM.

In several prior studies, investigators have used machine learning approaches for IDH prediction of gliomas. For example, Looze et al. (De Looze et al. 2018) devised a machine learning algorithm to determine a newly diagnosed glioma’s IDH status with an accuracy of 0.779, and ROC analysis yielded an AUC of 0.880 for the classification of IDH status in grade 2/3/4 gliomas. Chang et al. 2018 achieved an AUC of 0.950 for differentiating IDH mutation status in low- and high-grade gliomas by applying a residual convolutional neural network. Chen et al. (Chen et al. 2018) proposed a multi-label nonlinear classification model to predict both MGMT and IDH genotypes of patients with high-grade gliomas, resulting in AUCs of 0.787 and 0.886 respectively. In accordance with the latest WHO CNS5 classification, a recent study also developed a multiple gene prediction model incorporating mutual information of each genetic alteration in glioblastoma and grade 4 astrocytoma, IDH-mutant (Sohn et al. 2021). The authors demonstrated that IDH mutation status was predicted with the highest AUC of 0.967. In our study, the mean AUPRC of the top-rank models was 0.971 for manual VOI_3_ and 0.974 for clustering H_34_ respectively, similar to the performance of the previous study.

Subregion identification is a critical step in defining anatomically (or radiographically) meaningful localized zones for characterization of a glioma lesion (Cui et al. 2016; O'Connor et al. 2015). Image signal-wise segmentation relies on the subjective judgment of neuroradiologists who visually analyze the MRI signal intensity to delineate tumor subregions. It is a widely adopted method in clinical practice, as it leverages the expertise of neuroradiologists which is relatively straightforward to implement and interpret. However, it can be prone to inter-observer variability, as different radiologists may have different interpretations. It may fail to capture all the heterogeneity within the tumor and unable to identify subtle differences in tumor subpopulations. For example, manual identification is performed by radiologists with the assumption that MRI signal characteristics correlate with specific anatomical regions/tissues — enhancement on T_1_WI + C is typically considered a tumor entity and central non-enhancing hypointense signal represents necrosis. Nevertheless, global signal trends (e.g., enhancing, non-enhancing) usually define relatively large anatomical zones, and might not necessarily reflect morphological/pathological complexities within a much smaller scale (e.g., pixel-level). This hypothesis was supported by emerging evidence that high tumor cellularity is detected in both enhancing and non-enhancing regions of the GBM (Ye et al. 2020). The clustering algorithm has been used to characterize subregions —so-called “*habitats*”— that were pertinent to distinct subpopulations harboring divergent biological behaviors, which had therapeutic and prognostic implications (Fan et al. 2021; Shen et al. 2021; Zhang et al. 2021). Habitat imaging segmentation involves the analysis of the entire tumor volume and the identification of distinct subpopulations based on similarities in voxel characteristics, including intensity, texture, spatial location, and more. This approach allows for the capture of more detailed and nuanced information about tumor heterogeneity by exploring multiple voxel characteristics. Furthermore, it can identify subpopulations that may not be easily discernible based solely on signal intensity. Ultimately, this method has the potential to provide more objective and reproducible results compared to segmentation based solely on image signal. In this study, we compared the two aforementioned subregion definition strategies and the best performance was seen in clustering subregion H_34_ with all four sequences T_1_WI, T_2_WI, T_2_-FLAIR and T_1_WI + C fused in the RFO model. This suggests that voxel-based clustering subregions might also define heterogeneity-related intratumoral territories when reliable radiomics signatures are extracted.

The two optimal VOIs, manual VOI_3_ and clustering H_34,_ were composed of tumor peripheral edematous regions. This indicates that the edema area contains informative spatial diversity signatures associated with either molecular alterations or aggressive tumor behavior, both of which contribute to differentiating grade 4 astrocytoma from GBM. This corroborates tumor heterogeneity phenotypes manifesting in surrounding edematous regions (Dong et al. 2020; Li et al. 2018a, b). Information that was useful for discrimination was obtained from clustering H_4_. This subregion H_4_ (with low T_1_WI + C and low T_2_-FLAIR signals) presumably represents a region with low blood flow but high cell density. Such regions are correlated with potential treatment-resistant tumor cells that have adapted to the hypovascular tumor microenvironment (Gatenby et al. 2013). For example, a preliminary study found that GBM patients with poor prognosis had large subregions with low enhancement and relatively high cellularity, which is possibly due to compensatory adaptations of tumor cells in regions of poor vascularity, which result in either increased proliferation or utilization of substrates — due to Warburg physiology — to increase glucose uptake or toxic acid production in well-perfused regions (Zhou et al. 2014). Similarly, Stringfield et al. found that long-term GBM survivors had smaller subregions of low enhancement and high/low T_2_-FLAIR (corresponding to H_34_ in this study) than their short-term counterparts (Stringfield et al. 2019). Thus, both tumor edema and subregions with low T_1_WI + C and high/low T_2_-FLAIR are crucial for distinguishing the underlying genetic changes between grade 4 astrocytoma and GBM.

In this study, seven first-order statistics features, and three shape-based features were the top-performing radiomics signatures. In particular, the ‘Energy’, ‘Kurtosis’, ‘Mean’, ‘Median’, ‘RootMeanSquared’, ‘Skewness’ and ‘Maximum2DDiameterRow’ features exhibited statistically significant differences (*p* < 0.05) between the two groups. These imaging phenotypes may harbor underlying biological or genetic heterogeneity information in GBM patients (Park et al. 2021). For example, a higher degree of necrosis possibly explains the lower ‘mean’, ‘median’ and ‘RootMeanSquared’ values in GBM than in grade 4 astrocytoma, and hence the quantitative values characterizing the intensity distribution (e.g., ‘Energy’: measures the degree of intensity contained in a single bin in the histogram, ‘Kurtosis’ and ‘Skewness’: measures the peak height/width ratio and the symmetry of intensity distribution). Moreover, the grade 4 astrocytoma group had smaller ‘Maximum2DDiameterRow’ values than the GBM group (*p* = 0.018), indicating that tumors in the GBM are more stretched in the sagittal direction.

Several limitations should be addressed. First, the patient cohort size was limited due to its retrospective nature. However, the number of grade 4 astrocytoma patients (i.e., glioblastoma, IDH mutant in the 2016 standard) in our cohort corroborated their population prevalence (Figini et al. 2018). Second, according to the 2021 WHO CNS5, IDH wildtype diffuse astrocytic glioma (grade 2, 3) in adults with TERT promoter mutation, or EGFR gene amplification, or + 7/ − 10 chromosome copy number changes are now classified as GBM. However, only one case of the IDH wildtype diffuse astrocytoma with TERT promoter mutation and + 7/ − 10 copy number changes was included in our study due to a lack of routine molecular testing in our institution. Third, four clustering subregions were derived from T_1_WI + C and T_2_-FLAIR sequences. Whether more clustering subregions could achieve better performances is worth further investigation. Lastly, interpreting the generated clusters and assigning meaningful labels to the subregions could pose a greater challenge. Validating and correlating them with clinical outcomes would still be necessary to establish their clinical usefulness. While habitat imaging segmentation has the potential to capture more intricate tumor heterogeneity, it may demand additional computational resources and validation to prove its clinical efficacy. Further studies are required to generate insights that explain the links between clinical phenotypes (e.g., hypoxia and acidosis) and their radiographical phenotypes.

## Conclusion

In conclusion, subregions defined by clustering achieved discriminative accuracy comparable to manual delineation. Fusion of features from edematous subregions of multiple MRI sequences by the RFO model identified IDH genotypes of adult type grade 4 gliomas in line with current WHO CNS 5 criteria.

## Supplementary Information

Below is the link to the electronic supplementary material.

Supplementary file1 (DOCX 21 KB)

## Data Availability

The data sets used during the present study are available from the corresponding author upon reasonable request.

## Abbreviations

ACC: Accuracy
SEN: Sensitivity
SPE: Specificity
PRC: Precision-recall curve
AUPRC: Area under the precision-recall curve
GBM: Glioblastoma
IDH: Isocitrate dehydrogenase
WHO: World Health Organization
CNS: Central nervous system
T_1_WI + C: Contrast-enhanced T_1_WI
T_2_-FLAIR: T_2_‐weighted fluid-attenuated inversion recovery
SMOTE: Synthetic minority over-sampling technique
VOI: Volume of interests
MRI: Magnetic resonance imaging
nET: Non-enhanced tumor
ET: Enhanced tumor
pTE: Peritumoral edema
