# Supplementary material for: A subregion-based RadioFusionOmics model discriminates between grade 4 astrocytoma and glioblastoma on multisequence MRI
Source: J Cancer Res Clin Oncol. 2024 Feb 2;150(2):73. doi: 10.1007/s00432-023-05603-3 (PMC10837235; doi:10.1007/s00432-023-05603-3)
Supplement: Supplementary file 1 — Supplementary file1 (DOCX 21 KB) [file 432_2023_5603_MOESM1_ESM.docx]

**Supplementary materials**

*K-Means*:

For each Sample $N$, we extracted its voxels in the delineated VOI region on $c$ sequences ($c$ = 2) to get a sample set $D= \left\{ x_{1}, x_{2},\ldots,x_{m} \right\}$. Specify the number of cluster $k$ $\left\{ C_{1},C_{2},\ldots,C_{k} \right\}$ and select a set of values $\left\{ \mu_{1},\mu_{2},\ldots,\mu_{k} \right\} (k=2)$ as the initial centroid randomly to perform the below steps:

1). Compute the sum of the squared distance between $x_{i} (i=1,2,\ldots,m)$(except the centroid) and all centroid $\mu_{j} (j=1,2,\ldots,k)$: $d_{ij}= \left\| x_{i}- \mu_{j} \right\|_{2}^{2}$. If $d_{ij}$ is the smallest of all centroid, put $x_{i}$ into the category $\lambda_{j}$ corresponding to $\mu_{j}$ and update $C_{\lambda_{j}}= C_{\lambda_{j}}\cup\{x_{i}\}$

2). Recalculate the new centroid based on the new $C_{j} (j=1,2,\ldots,k)$ by $\mu_{j}=\frac{1}{C_{j}}\sum_{x\in C_{j}} x$

Repeat steps 1) and 2) until all centroids$\mu$ are no longer change, and output the final clustering result $C=\left\{ C_{1},C_{2},\ldots,C_{k} \right\}$. By far, we divided all voxels in the VOI region into high and low, and combine them into $2^{c}$ regions.

*Feature Fusion:*

Firstly, a feature matrix $X_{(p\times n)}$ is defined, which $p$ ($p$ = 2,3 or 4) represent the number of MRI sequences and $n$ is the training sample size. In order to incorporate the class of the training data to the feature fusion, $n$ columns of matrix $X$ were separated into $c$ groups ($c$ is the number of classes, $c$ = 2), where $n_{i}$ columns belong to the *i*th class ($n = \sum_{i=1}^{c} n_{i}$). The sample $x_{i,j}$ belonging to $X$ represent the *j*th sample in the *i*th class, and $\overline{x}_{i} (\overline{x}_{i} = \frac{1}{n_{i}}\sum_{j=1}^{n_{i}} x_{i,j})$ and $\overline{x} (\overline{x} = \frac{1}{n}\sum_{i=1}^{c} n_{i}\overline{x}_{i})$ represent the mean of the *i*th class and the whole data individually. Then, a inter-class scatter matrix $S_{(p\times p)} = \sum_{i=1}^{c} n_{i}{(\overline{x}_{i}-\overline{x})(\overline{x}_{i}-\overline{x})}^{T} = \Phi\Phi^{T}$ is constructed, where $\Phi=[ \sqrt{n_{1}}(\overline{x}_{1}-\overline{x}), \sqrt{n_{2}}(\overline{x}_{2}-\overline{x}),...,\sqrt{n_{c}}(\overline{x}_{c}-\overline{x})]$. If the classes were well-separated, the covariance matrix $\Phi\Phi^{T}$ would be a diagonal matrix, and since it is symmetric positive semidefinite, it can be diagonalized as $P^{T}\Phi\Phi^{T}P = \Lambda$, where $P$ is the matrix of orthogonal eigenvectors and $\Lambda$ is the diagonal matrix of real non- negative eigenvalues sorted in decreasing order. The first r eigenvectors selected from P were made to the matrix $Q_{(c\times r)}$, then ${(\Phi Q)}^{T}S(\Phi Q) =\Lambda_{(r\times r)}$ can be formulated with mapping Q to $\Phi Q$. Therefore, the matrix $W_{(p\times r)} =\Phi Q\Lambda^{-\frac{1}{2}}$ unitizes S by $W^{T}SW = I$ and reduces the dimension of matrix X from $p$ to $r$. Feature fusion can be done by setting $r = 1$ to compress the matrix $X_{(p\times n)}$ to a row vector $x_{(1\times n)}$ ($x_{(1\times n)} = W_{(1\times p)}^{T}X_{(p\times n)}$).

*Acquisition parameters:*

Axial T1-weighted image (T_1_WI) (repetition time/echo time (TR/TE), 400~2000/9~15 ms; section thickness, 6 mm; number of signals acquired, 2; matrix, 320 _ 320; field of view (FOV), 230 _ 230 mm), axial T_2_-weighted image (T_2_WI) ((TR/TE, 3500~6000/95~100 ms; section thickness, 6 mm; number of signals acquired, 1; matrix, 320 _ 320; FOV, 230 _ 230 mm), and axial T_2_-weighted fluid attenuated inversion recovery (T_2__FLAIR) images ((TR/TE, 6000~8000/90~120 ms; section thickness, 6 mm; number of signals acquired, 1; matrix, 320 _ 320; FOV, 230 _ 230 mm)).

*RadioFusionOmics:*

RadioFusionOmics(RFO) methodology is a novel two-level fusion scheme that integrates radiomics information from different MRI sequences. It exploits the ensemble learning to fully explore the strengths of various classifiers. First, the $4\times n\times p$ feature matrix ($n$ represents the number of patients and $p$ is the number of features) extracted from the 4 MRI sequences of all patients is used to obtain a $n\times p$ fused feature matrix and a transformation matrix (the feature matrix used to fuse the independent test set) by the feature fusion method in Page 01 Line 15. Next, the fused feature matrix is applied into the model fusion stage(4 classifiers and 6 feature selection algorithms), and the best model combination is selected by ranking the PRAUC. The chosen model will be applied to the transformed test set.
